# Tenascin-C, presepsin (SCD14-ST), and neutrophil-to-lymphocyte ratio as complementary circulating biomarkers for sepsis-associated organ dysfunction and 28-day all-cause mortality: A biomarker-enriched prognostic model

**DOI:** 10.5937/jomb0-64095

**Published:** 2026-05-22

**Authors:** Yuxiang Xie, Chengzhi Xie

**Affiliations:** 1 The Second Affiliated Hospital of Hainan Medical University, Intensive Care Unit Ward 1, Haikou, China

**Keywords:** tenascin-C, presepsin (sCD14-ST), sepsis-associated organ dysfunction, biomarker-based risk stratification, 28-day all-cause mortality, tenascin-C, presepsin (sCD14-ST), disfunkcija organa povezana sa sepsom, stratifikacija rizika zasnovana na biomarkerima, mortalitet od svih uzroka u roku od 28 dana

## Abstract

**Background:**

Sepsis is characterized by dysregulated host responses with subsequent organ dysfunction. Conventional biomarkers (e.g., procalcitonin) incompletely reflect tissue injury and immune activation. We evaluated the clinical laboratory value of circulating Tenascin-C (TNC), presepsin (sCD14-ST), and the neutrophil-to-lymphocyte ratio (NLR) as complementary biomarkers of organ dysfunction severity and short-term prognosis in sepsis.

**Methods:**

This retrospective observational study enrolled 103 adult ICU patients fulfilling Sepsis-3 criteria (June 2023-June 2025). Peripheral blood was collected within 12 h of ICU admission. Serum TNC was measured by ELISA; sCD14-ST was quantified by chemiluminescent enzyme immunoassay; NLR was calculated from routine blood counts. Associations with organ dysfunction were assessed using Spearman correlation with SOFA scores. Independent associations with 28-day all-cause mortality were examined using multivariable logistic regression. Predictive performance of a clinical baseline model (age, SOFA, PCT) versus a biomarker-enriched model (baseline + TNC, sCD14-ST, NLR) was evaluated by ROC analysis.

## Abstract (sr)

**Uvod:**

Sepsu karakterišu disregulisani odgovori domaćina sa naknadnom disfunkcijom organa. Konvencionalni biomarkeri (npr. prokalcitonin) ne odražavaju potpuno oštećenje tkiva i imunološku aktivaciju. Procenili smo kliničku laboratorijsku vrednost cirkulišućeg tenascina-C (TNC), presepsina (sCD14-ST) i odnosa neutrofila i limfocita (NLR) kao komplementarnih biomarkera težine disfunkcije organa i kratkoročne prognoze kod sepse.

**Metode:**

Ova retrospektivna opservaciona studija obuhvatila je 103 odrasla pacijenta na intenzivnoj nezi koji su ispunjavali kriterijume za sepsu-3 (jun 2023 - jun 2025). Periferna krv je prikupljena u roku od 12 sati od prijema na intenzivnu negu. Serumski TNC je meren ELISA metodom; sCD14-ST je kvantifikovan hemiluminiscentnim enzimskim imunotestom; NLR je izračunat iz rutinske krvne slike. Povezanosti sa disfunkcijom organa procenjene su korišćenjem Spirmanove korelacije sa SOFA rezultatima. Nezavisne povezanosti sa 28-dnevnim mortalitetom od svih uzroka ispitane su korišćenjem multivarijantne logističke regresije. Prediktivne performanse kliničkog osnovnog modela (starost, SOFA, PCT) u odnosu na model obogaćen biomarkerima (početna vrednost + TNC, sCD14-ST, NLR) procenjene su ROC analizom.

**Rezultati:**

TNC, sCD14-ST i NLR bili su viši kod septičkog šoka nego kod sepse i bili su povišeni kod nepreživelih u odnosu na preživele (svi P &lt; 0,05). TNC je pokazao najjaču korelaciju sa SOFA (r = 0,466, P &lt; 0,001), zatim sCD14-ST (r = 0,352, P &lt; 0,001) i NLR (r = 0,299, P = 0,002). U multivarijantnoj analizi, TNC, sCD14-ST i NLR su ostali nezavisno povezani sa smrtnošću od svih uzroka u roku od 28 dana. Model obogaćen biomarkerima poboljšao je diskriminaciju u poređenju sa osnovnim modelom (AUC 0,928 naspram 0,867), sa većom osetljivošću (84,4% naspram 71,9%).

**Zaključak:**

Prijemni TNC, sCD14-ST i NLR obuhvataju komplementarne biohemijske dimenzije sepse (povređivanje tkiva, urođena imunološka aktivacija i imuni disbalans) i poboljšavaju stratifikaciju rizika za disfunkciju organa i 28-dnevni mortalitet od svih uzroka kada se dodaju rutinskim kliničkim varijablama. Ovi nalazi podržavaju pragmatičan, laboratorijski zasnovan multimarkerski pristup za ranu prognostičku procenu kod sepse.

## Introduction

Sepsis is a life-threatening organ dysfunction caused by a dysregulated host response to infection. Despite continuous advances in critical care medicine, sepsis remains one of the leading causes of mortality worldwide, accounting for approximately 11 million deaths annually [1]. Although short-term survival has improved in recent years, emerging epidemiological evidence indicates that the long-term disease burden associated with sepsis and its complications among ICU survivors remains substantial [2]. The rapid progression of sepsis to multiple organ dysfunction syndrome (MODS) underscores the importance of early identification of patients at high risk for organ failure, which is essential for timely intervention and improved survival.

Currently, conventional biomarkers such as C-reactive protein (CRP) and procalcitonin (PCT) are widely used for the diagnosis and monitoring of infection. However, these markers primarily reflect systemic inflammation and infection burden and have limited ability to accurately capture the extent of structural organ damage or to predict clinical outcomes [3]. A recent review by Chen et al. [4] emphasized the urgent need for novel biomarkers that bridge the gap between »systemic inflammation«and »organ-specific injury« in sepsis.

Tenascin-C is a large extracellular matrix glycoprotein that is minimally expressed in most normal adult tissues but is rapidly and transiently upregulated in response to tissue injury, hypoxia, and inflammation. As a damage-associated molecular pattern (DAMP), Tenascin-C can bind to Toll-like receptor 4 (TLR4) and activate downstream proinflammatory signaling pathways, thereby amplifying cytokine release and perpetuating a vicious cycle of inflammation and tissue injury [5][6]. Recent mechanistic studies have further demonstrated that activation of the TLR4/nuclear factor-kB (NF-kB) signaling pathway by Tenascin-C represents a critical step in driving inflammatory cascades [7]. Accumulating clinical evidence suggests that elevated Tenascin-C levels are closely associated with disease severity and poor prognosis in patients with sepsis [6][8].

Soluble CD14 subtype (sCD14-ST), also known as presepsin, is a specific biomarker of bacterial infection that reflects activation of monocytes and macrophages during innate immune responses [9][10]. Unlike PCT, which is mainly released by parenchymal cells in response to inflammatory cytokines, sCD14-ST is generated through lysosomal cleavage of membrane-bound CD14 during phagocytosis of pathogens, allowing it to more directly reflect immune activation and pathogen burden. The neutrophil-to-lymphocyte ratio (NLR), a readily available marker derived from routine blood tests, reflects the balance between innate immune activation and adaptive immune suppression and has been consistently associated with mortality in patients with sepsis [11][12].

Although Tenascin-C, sCD14-ST, and NLR represent distinct but complementary pathophysiological dimensions - namely tissue injury, infection burden, and immune status - few studies have systematically evaluated their combined clinical utility in sepsis. Therefore, this study aimed to investigate the associations between these biomarkers and Sequential Organ Failure Assessment (SOFA) scores, as well as their predictive value for 28-day all-cause mortality in patients with sepsis. By integrating biomarkers reflecting different aspects of sepsis pathophysiology, we sought to provide a more comprehensive and clinically useful tool for early risk stratification.

## Materials and methods

### Study design and participants

This retrospective observational study was approved by the Medical Ethics Committee of our hospital. Patients with sepsis who were admitted to the general intensive care unit (ICU) of our hospital between June 2023 and June 2025 were consecutively enrolled.

The inclusion criteria were as follows: (1) Diagnosis of sepsis according to the Sepsis-3 criteria [13]. Sepsis was defined as life-threatening organ dysfunction caused by a dysregulated host response to infection. Organ dysfunction was identified as an increase in the Sequential Organ Failure Assessment (SOFA) score of ≥2 points from baseline in patients with suspected infection. (2) Age ≥ 18 years.

The exclusion criteria were as follows: (1) Age < 18 years; (2) Pregnancy; (3) Presence of malignant tumors, autoimmune diseases, or pre-existing chronic organ dysfunction, including chronic kidney disease stage ≥3, New York Heart Association (NYHA) class 111—IV heart failure, or Child-Pugh class C liver cirrhosis; (4) History of major surgery or severe trauma within 2 weeks prior to admission; (5) Use of systemic glucocorticoids or other immunosuppressive agents within 2 weeks prior to admission; (6) Hematological disorders affecting leukocyte counts; (7) Missing key clinical data (age, site of infection, SOFA score) or core laboratory parameters.

A total of 103 patients were ultimately included, of whom 67 were assigned to the sepsis group and 36 to the septic shock group. Septic shock was defined according to the Sepsis-3 criteria [13] as sepsis with persistent hypotension requiring vasopressors to maintain a mean arterial pressure (MAP) ≥ 65 mmHg despite adequate fluid resuscitation, along with a serum lactate level > 2 mmol/L. In the absence of clear clinical evidence of tissue hypoperfusion, lactate levels were re-measured within 2-4 hours for confirmation. All patients received standardized treatment strategies, including early fluid resuscitation, goal-directed therapy, broad-spectrum antimicrobial therapy, and organ function support as clinically indicated.

**Figure 1:**
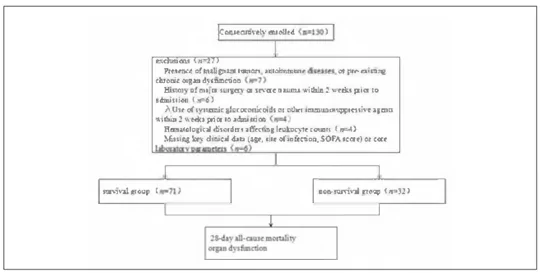
The patient selection flow chart.

The patient selection flow chart is shown in (Figure 1).

### Data collection

Demographic data (age and sex), comorbidities (including hypertension and diabetes mellitus), vital signs (body temperature, heart rate, and blood pressure), site of infection, and source of ICU admission were collected at admission. Disease severity was assessed using the Acute Physiology and Chronic Health Evaluation II (APACHE II) score and the SOFA score.

### 28-day all-cause mortality

Patient 28-day all-cause mortality was obtained via the electronic medical record (EMR) system. Based on 28-day all-cause mortality, patients were divided into a survival group (n = 71) and a non-survival group (n = 32).

### Laboratory measurements

Peripheral venous blood samples (5 mL) were collected from all patients within 12 hours of ICU admission and processed in two tubes. One sample was collected into a vacuum tube without anticoagulant, allowed to stand at room temperature for 30 minutes, and centrifuged at 3500 rpm for 10 minutes (centrifugation radius: 8 cm; temperature: 4°C). The supernatant serum was separated, aliquoted into sterile 1.5 mL microcentrifuge tubes, and stored at -80°C for subsequent analysis of Tenascin-C (TNC) and C-reactive protein (CRP). The second sample was collected into an EDTA-anticoagulated vacuum tube, gently inverted five times, and used for routine blood counts, procalcitonin (PCT), lactate, and soluble CD14 subtype (sCD14-ST) measurements.

Serum Tenascin-C levels were measured using a commercial enzyme-linked immunosorbent assay (ELISA) kit (R&D Systems, USA; catalog no. NBP2-78766) in strict accordance with the manufacturer's instructions. Serum sCD14-ST concentrations were determined using a chemiluminescent enzyme immunoassay on a fully automated analyzer (PATH-FAST; Mitsubishi Chemical Medience, Japan) with a matching reagent kit (catalog no. 94001). From blood sampling/enrollment to biomarker measurement, the median storage time was 1 hour (maximum, 2 hours). All samples were assayed at the first thaw, with ≤1 freeze-thaw cycle. Samples were stratified by outcome and then randomly allocated across plates/batches to ensure similar group proportions within each batch. Whenever possible, assays were completed using the same kit lot to minimize between-batch drift. Each plate included two levels (high and low) of pooled serum quality-control (QC) materials prepared from the same batch and aliquoted for frozen storage to monitor inter-batch stability; if QC results exceeded prespecified thresholds, the entire plate was repeated or re-evaluated. When a new reagent lot was introduced, the master calibration curve was installed and a user calibration was performed, with calibrators measured in duplicate, and routine QC was conducted according to the instrument/manufacturer's instructions. The overall assay imprecision (total CV) was 4.1%-5.0%.

NLR was calculated as the ratio of absolute neutrophil count to absolute lymphocyte count obtained from routine blood analysis using an automated hematology analyzer (Sysmex XN-9000).

PCT levels were measured using an immuno-chromatographic assay on a fully automated immunoassay analyzer (Cobas e411; Roche Diagnostics), with a detection range of 0.02-100 ng/mL and a lower detection limit of 0.02 ng/mL. CRP concentrations were measured by immunoturbidimetry using an automated biochemical analyzer (AU5800; Beckman Coulter), with a detection range of 0.1-200 mg/L. Serum lactate levels were determined using the lactate dehydrogenase method on the same analyzer, with a detection range of 0.5-20 mmol/L. All laboratory measurements were performed in accordance with standardized operating procedures, and instruments were regularly calibrated with routine quality control.

### Statistical analysis

Statistical analyses were performed using SPSS software version 26.0 (IBM Corp., Armonk, NY, USA). Data normality was assessed using the Shapiro-Wilk test. Continuous variables with non-normal distribution were expressed as medians with interquartile ranges (IQR) and compared using the Mann-Whitney U test. Categorical variables were presented as numbers and percentages and compared using the c^2^ test. Spearman's rank correlation coefficient was used to analyze correlations between biomarkers and SOFA scores. To identify potential influencing factors associated with 28-day all-cause mortality, logistic regression analysis was performed in this study based on previous clinical research and available data from the present clinical study.In this study, EPV = 32/6 ≈ 5.3 > 5, meeting the minimum sample size requirement for logistic regression analysis. Multivariate logistic regression models were built to evaluate the incremental predictive value of adding Tenascin-C, sCD14-ST, and NLR to the clinical baseline model for predicting 28-day all-cause mortality. Prior to logistic regression analysis, collinearity diagnostics were performed, with a variance inflation factor (VIF) threshold of 10. Receiver operating characteristic (ROC) curves were constructed, and the area under the curve (AUC), 95% confidence intervals (95% CI), optimal cutoff values, sensitivity, and specificity were calculated. The areas under the curves were compared using the Delong test, and internal validation was performed with Bootstrap (1,000 replications). A two-sided P value < 0.05 was considered statistically significant.

## Results

### Baseline characteristics and comparison between survivors and non-survivors

A total of 103 patients with sepsis were included in the final analysis. The 28-day survival rate was 68.9% (71/103), and the 28-day all-cause mortality rate was 31.1% (32/103). The SOFA score was significantly higher in non-survivors than in survivors (11.50 (10.00-13.00) vs. 8.00 (6.00-9.00), P < 0.001).

**Figure 2:**
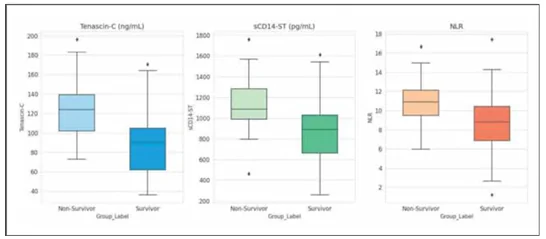
Comparison of serum Tenascin-C, sCD14-ST, and NLR levels between survivors and non-survivors.

With respect to biomarkers, serum levels of Tenascin-C, sCD14-ST, and NLR were all significantly elevated in the non-survival group compared with the survival group (all P < 0.001). Among traditional inflammatory markers, procalcitonin (P = 0.026) and lactate (P = 0.008) levels were significantly higher in non-survivors, whereas CRP levels did not differ significantly between the two groups (P = 0.201). Detailed baseline characteristics and laboratory findings are summarized in Table 1. Group comparisons of Tenascin-C, sCD14-ST, and NLR are illustrated in Figure 2.

**Table 1:** Baseline characteristics and laboratory parameters of survivors and non-survivors.

Variables	Survivors (n = 71)	Non-survivors (n = 32)	c^2^ / t / Z	P value
Age (years)	52.66 ± 10.05	52.28 ± 10.28	0.177	0.860
Male, n (%)	43 (60.56)	21 (65.63)	0.240	0.624
Comorbidities, n (%)				
Hypertension	28 (39.44)	14 (43.75)	0.170	0.680
Diabetes mellitus	19 (26.76)	11 (34.38)	0.620	0.431
Body mass index (kg/m^2^)	23.42 ± 3.15	24.08 ± 3.67	0.455	0.651
Site of infection, n (%)				
Pulmonary	35 (49.30)	18 (56.25)	0.427	0.513
Abdominal	16 (22.54)	8 (25.00)	0.075	0.784
Urinary tract	9 (12.68)	5 (15.63)	0.163	0.686
Source of admission, n (%)			0.101	0.951
Emergency department	45 (63.38)	20 (62.50)		
Postoperative	16 (22.54)	8 (25.00)		
Medical ward transfer	10 (14.08)	4 (12.50)		
Vital signs				
Heart rate (beats/min)	92.52 ± 15.37	98.68 ± 18.72	1.630	0.190
Mean arterial pressure (mmHg)	78.46 ± 8.62	76.36 ± 10.15	1.017	0.314
Body temperature (°C)	37.56 ± 0.65	37.68 ± 0.71	0.815	0.419
Severity scores				
SOFA score	8.00 (6.00-9.00)	11.50 (10.00-13.00)	5.899	<0.001
APACHE II score	17.00 (14.50-20.00)	17.50 (15.75-20.00)	0.583	0.560
Use of vasopressors (vasoactive agents), n (%)	33 (46.48)	14 (43.75)	0.066	0.797
Mechanical ventilation, n (%)	28 (39.44)	15 (46.88)	0.502	0.479
CRRT, n (%)	36 (50.70)	21 (65.63)	1.987	0.159
Biomarkers				
Tenascin-C (ng/m L)	90.25 (61.77-104.89)	123.87 (101.86-139.19)	5.095	<0.001
sCD 14-ST (pg/m L)	889.30 (664.30-1028.13)	1085.16 (989.77-1282.94)	4.062	<0.001
NLR	8.80 (6.88-10.44)	10.91 (9.49-12.12)	3.692	<0.001
PCT (ng/m L)	6.69 (5.64-8.57)	8.36 (6.48-9.36)	2.221	0.026
Lactate (mmol/L)	2.65 (1.94-3.29)	3.52 (2.25-4.22)	2.644	0.008
CRP (mg/L)	41.78 (34.44-48.45)	44.62 (37.45-48.78)	1.279	0.201

### Comparison of biomarkers according to disease severity

Patients in the septic shock group exhibited significantly higher serum levels of Tenascin-C, sCD14-ST, and NLR compared with those in the sepsis group (all P < 0.05), indicating that these biomarkers increased with disease severity (Table 2).

**Table 2:** Comparison of biomarkers between sepsis and septic shock groups.

Variables	Sepsis (n = 67)	Septic shock (n = 36)	Z	P value
Tenascin-C (ng/m L)	90.25 (61.77-105.66)	116.59 (95.68-139.19)	4.337	<0.001
sCD 14-ST (pg/m L)	889.30 (673.85-1021.05)	1060.28 (902.07-1286.80)	3.828	<0.001
NLR	8.99 (6.93-10.90)	10.41 (8.64-12.05)	2.144	0.032

### Correlations between biomarkers and SOFA scores

**Figure 3:**
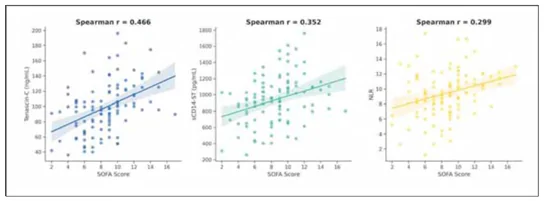
Spearman correlation analysis between Tenascin-C, sCD14-ST, NLR, and SOFA scores.

Spearman correlation analysis demonstrated that Tenascin-C showed the strongest positive correlation with SOFA score (r = 0.466, P < 0.001). sCD14-ST was moderately correlated with SOFA score (r = 0.352, P < 0.001), while NLR exhibited a weaker but significant positive correlation (r = 0.299, P = 0.002) (Figure 3).

### Independent risk factors for 28-day all-cause mortality

Before conducting logistic regression analysis, collinearity diagnostics were performed. The variance inflation factor (VIF) of each indicator was <10, indicating no significant multicollinearity among the variables. In Model 1, which included only clinical baseline variables (age, SOFA score, and PCT), SOFA score was identified as an independent risk factor for 28-day all-cause mortality (P < 0.05). After adding Tenascin-C, sCD14-ST, and NLR to the baseline model (Model 2), Delong test showed that the explanatory power of the model was substantially improved (Table 3). All three biomarkers were independently associated with 28-day all-cause mortality.

**Table 3:** Multivariate logistic regression analysis for 28-day mortality in patients with sepsis. Note: Variables were rescaled prior to analysis.

Variables	b	S.E.	Wald	P value	OR (95% CI)
Model 1: Clinical baseline model					
Age	-0.014	0.027	0.283	0.595	0.986 (0.935-1.040)
SOFA score	0.649	0.142	20.747	0.001	1.913 (1.447-2.529)
PCT	0.165	0.125	1.749	0.186	1.180 (0.923-1.507)
Model 2: Biomarker-enriched model					
Age	-0.033	0.037	0.804	0.370	0.967 (0.899-1.040)
SOFA score	0.448	0.142	9.944	0.002	1.566 (1.185-2.069)
PCT	0.078	0.141	0.307	0.579	1.081 (0.820-1.426)
Tenascin-C (per 10 ng/mL)	0.230	0.120	4.070	0.044	1.268 (1.001-1.583)
sCD 14-ST (per 100 pg/mL)	0.300	0.100	4.428	0.035	1.349 (1.105-1.647)
NLR (per 5 units)	1.495	0.640	5.492	0.019	4.459 (1.276-15.548)

### Predictive performance of biomarkers for 28-day all-cause mortality

**Figure 4:**
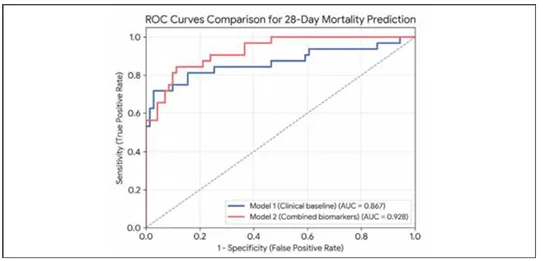
Receiver operating characteristic (ROC) curves for predicting 28-day mortality using the clinical baseline model and the biomarker-enriched model.

**Figure 5:**
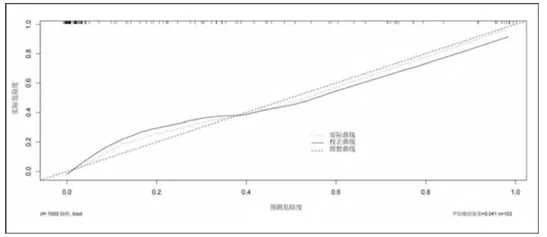
Calibration curve.

**Figure 6:**
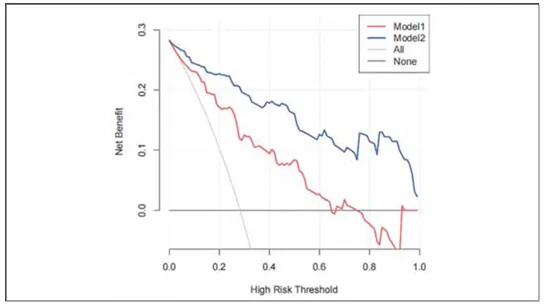
Decision curve analysis.

ROC curve analysis showed that the AUC of Model 1 was 0.867 (95% CI: 0.765-0.952), with a sensitivity of 71.9% and a specificity of 97.2%. After incorporation of Tenascin-C, sCD14-ST, and NLR (Model 2), the AUC increased to 0.928 (95% CI: 0.873-0.974), with a sensitivity of 84.4% and a specificity of 88.7%, indicating a significant improvement in predictive performance (Figure 4, Figure 5, Figure 6, Table 4). Internal validation using Bootstrap with 1,000 replications showed an AUC of 0.907, indicating that the model was stable.

**Table 4:** Predictive performance of biomarkers for 28-day all-cause mortality.

Variables	AUC	95% CI	Youden index	sensitivity %	specificity %	optimal cutoffs
Age	0.604	0.518~0.751	0.372	69.75	56.76	52
SOFA score	0.679	0.641~0.782	0.441	80.83	72.51	11
PCT	0.661	0.594~0.742	0.402	68.52	79.70	7.53 ng/mL
Tenascin-C (per 10 ng/mL)	0.704	0.659~0.791	0.469	72.85	83.83	103.19 ng/mL
sCD 14-ST (per 100 pg/mL)	0.711	0.643~0.856	0.473	76.39	75.65	1018.74 pg/m L
NLR (per 5 units)	0.708	0.617~0.796	0.470	75.01	69.85	10.04
Model 1	0.867	0.765~0.952	0.691	71.9	97.2	/
Model 2	0.928	0.873~0.974	0.731	84.4	88.7	/

## Discussion

Sepsis is a complex and heterogeneous syndrome characterized by infection-induced immune dysregulation and subsequent organ dysfunction. Although biomarkers such as procalcitonin (PCT) and C-reactive protein (CRP) are widely used in clinical practice, they primarily reflect systemic inflammation or infection burden and have limited ability to accurately assess the extent of organ injury or predict prognosis. In this study, we systematically evaluated the clinical value of three biomarkers representing distinct pathophysiological dimensions—Tenascin-C (tissue injury), sCD14-ST (infection and innate immune activation), and neutrophil-to-lymphocyte ratio (NLR; immune status)—in the assessment of organ dysfunction and 28-day all-cause mortality in patients with sepsis.

The main findings of this study are as follows: (1) serum Tenascin-C, sCD14-ST, and NLR levels were positively correlated with SOFA scores and increased with disease severity; (2) all three biomarkers were independent risk factors for 28-day all-cause mortality; and (3) a combined biomarker model incorporating Tenascin-C, sCD14-ST, and NLR significantly improved prognostic accuracy compared with a clinical baseline model alone (AUC = 0.928).

### Tenascin-C and organ dysfunction in sepsis

Tenascin-C is an extracellular matrix glycoprotein that is minimally expressed in healthy adult tissues but is rapidly induced under conditions of tissue injury, hypoxia, and inflammation [5]. As a damage-associated molecular pattern (DAMP), Tenascin-C plays an active role in amplifying inflammatory responses rather than serving merely as a byproduct of tissue damage. Mechanistically, Tenascin-C contains a fibrinogen-like globe (FBG) domain that functions as an endogenous ligand for Toll-like receptor 4 (TLR4), leading to sustained activation of the NF-kB signaling pathway and enhanced production of proinflammatory cytokines such as interleukin-6 and tumor necrosis factor-a [6][7].

In the present study, serum Tenascin-C levels were significantly higher in non-survivors than in survivors and showed the strongest correlation with SOFA scores among the evaluated biomarkers (r = 0.466). These findings suggest that Tenascin-C may more directly reflect the extent of sepsis-induced structural organ damage rather than merely the systemic inflammatory response. Consistent with our results, previous studies have reported that elevated Tenascin-C levels are associated with disease severity and adverse outcomes in patients with sepsis [6][8]. Our findings further support the concept that a self-amplifying »Tenascin-C-TLR4-inflammatory cascade« may contribute to persistent inflammation and progressive organ dysfunction in sepsis, although experimental validation of this mechanism in the context of sepsis is still warranted [14].

### sCD14-ST as a marker of infection burden and innate immune activation

Soluble CD14 subtype (sCD14-ST), also known as presepsin, is released during monocyte/macrophage-mediated phagocytosis of pathogens through enzymatic cleavage of membrane-bound CD14 [9]. Unlike PCT, which is largely produced by parenchymal cells in response to inflammatory cytokines and may exhibit delayed elevation, sCD14-ST directly reflects activation of the innate immune system and pathogen load [15][16]. This biological distinction may explain its superior performance in certain clinical scenarios.

In this study, sCD14-ST levels were significantly higher in non-survivors and in patients with septic shock, and multivariate analysis confirmed sCD14-ST as an independent predictor of 28-day all-cause mortality. Furthermore, sCD14-ST was moderately correlated with SOFA scores (r = 0.352), indicating that it may be linked not only to infection severity but also to the progression of organ dysfunction. These findings are consistent with a recent systematic review by Xu et al. [17], which highlighted the close association between immune activation biomarkers and organ dysfunction in sepsis. Compared with PCT, sCD14-ST may offer advantages in early risk stratification, as its levels rise earlier in the course of infection and appear to be less influenced by prior antibiotic therapy.

### NLR and immune dysregulation in sepsis

The neutrophil-to-lymphocyte ratio is a simple and readily available marker that reflects the balance between innate immune activation and adaptive immune suppression. Neutrophilia represents excessive activation of the innate immune response, whereas lymphopenia is a hallmark of sepsis-induced immunosuppression. In the present study, NLR was significantly higher in non-survivors and was identified as an independent predictor of 28-day all-cause mortality.

These findings are supported by growing evidence from large-scale studies and meta-analyses. Gao et al. [18], in a meta-analysis involving over 23,000 patients, demonstrated a strong association between elevated NLR and increased mortality in sepsis. Similarly, analyses based on large critical care databases have confirmed the robustness of NLR in predicting short-term mortality [19]. From a pathophysiological perspective, elevated cortisol levels and catecholamine surges during sepsis can induce lymphocyte apoptosis, thereby impairing adaptive immune defenses and increasing susceptibility to secondary infections. As a composite index, NLR captures these dynamic changes more sensitively than absolute leukocyte counts alone.

### Clinical implications of combined biomarker assessment

Sepsis involves multiple interconnected pathophysiological processes, including pathogen invasion, dysregulated immune responses, and secondary organ injury. The combined assessment strategy proposed in this study integrates biomarkers reflecting tissue injury (Tenascin-C), infection burden and innate immune activation (sCD14-ST), and host immune status (NLR), thereby providing a more comprehensive evaluation of disease severity.

ROC curve analysis demonstrated that the combined biomarker model achieved an AUC of 0.928 for predicting 28-day all-cause mortality, outperforming the clinical baseline model. This multimodal biomarker approach may serve as a useful adjunct for early risk stratification and clinical decision-making. Patients with simultaneous elevations in Tenascin-C, sCD14-ST, and NLR may represent a subgroup with severe infection, profound immune dysregulation, and ongoing organ damage, warranting intensified monitoring, aggressive organ support, and consideration of immunomodulatory interventions.

### Limitations

Several limitations of this study should be acknowledged. First, this was a single-center retrospective study with a relatively small sample size, which may limit the generalizability of the findings. Second, only biomarker levels within the first 12 hours of ICU admission were analyzed; dynamic changes and clearance rates of these biomarkers may provide additional prognostic information. Third, this study did not include analyses targeting specific organ systems, and future studies should explore the relationships between these biomarkers and individual organ dysfunction trajectories. Finally, this exploratory analysis did not apply adjustments for multiple comparisons, and the results should therefore be interpreted with caution.

## Conclusions

In conclusion, serum Tenascin-C, sCD14-ST, and neutrophil-to-lymphocyte ratio are valuable biomarkers for assessing disease severity and prognosis in patients with sepsis. Among them, Tenascin-C is closely associated with organ dysfunction and may reflect the extent of sepsis-induced tissue injury. All three biomarkers were identified as independent predictors of 28-day all-cause mortality. Combined assessment of Tenascin-C, sCD14-ST, and NLR significantly improves prognostic accuracy and may facilitate early identification of high-risk patients, thereby supporting individualized treatment strategies and optimized clinical decision-making in sepsis management.

## Dodatak

### Conflict of interest statement

All the authors declare that they have no conflict of interest in this work.
